# TGFβ Family Signaling Pathways in Pluripotent and Teratocarcinoma Stem Cells’ Fate Decisions: Balancing Between Self-Renewal, Differentiation, and Cancer

**DOI:** 10.3390/cells8121500

**Published:** 2019-11-23

**Authors:** Olga Gordeeva

**Affiliations:** Kol’tsov Institute of Developmental Biology, Russian Academy of Sciences, 26 Vavilov str., 119334 Moscow, Russia; olgagordeeva@yandex.ru; Tel.: +7-499-135-8780

**Keywords:** TGFbeta, BMP, cell signaling, cancer stem cells, pluripotent stem cells, teratocarcinoma

## Abstract

The transforming growth factor-β (TGFβ) family factors induce pleiotropic effects and are involved in the regulation of most normal and pathological cellular processes. The activity of different branches of the TGFβ family signaling pathways and their interplay with other signaling pathways govern the fine regulation of the self-renewal, differentiation onset and specialization of pluripotent stem cells in various cell derivatives. TGFβ family signaling pathways play a pivotal role in balancing basic cellular processes in pluripotent stem cells and their derivatives, although disturbances in their genome integrity induce the rearrangements of signaling pathways and lead to functional impairments and malignant transformation into cancer stem cells. Therefore, the identification of critical nodes and targets in the regulatory cascades of TGFβ family factors and other signaling pathways, and analysis of the rearrangements of the signal regulatory network during stem cell state transitions and interconversions, are key issues for understanding the fundamental mechanisms of both stem cell biology and cancer initiation and progression, as well as for clinical applications. This review summarizes recent advances in our understanding of TGFβ family functions in naїve and primed pluripotent stem cells and discusses how these pathways are involved in perturbations in the signaling network of malignant teratocarcinoma stem cells with impaired differentiation potential.

## 1. Introduction

The transforming growth factor-β (TGFβ) family factors are involved in the regulation of most normal and pathological cellular processes from early development, morphogenesis and histogenesis to various metabolic diseases and cancers. More than 40 factors of the TGFβ family support a dynamic equilibrium of processes during the normal functioning of various cells. TGFβ family factors can activate several signaling pathways that induce pleiotropic and even antagonistic effects when they interact with other signaling pathways [1,2].

Maintaining a balance between proliferation, differentiation, and cell death in stem cells is the basis of self-renewing tissue homeostasis. The activity of different branches of TGFβ family signaling pathways and their interactions with other signaling pathways and intracellular targets govern the fine regulation of the self-renewal, differentiation onset, and step-by-step specialization processes of stem cell descendants in various cell derivatives [3,4]. However, pluripotent stem cells in the early embryo, like multipotent stem cells in adult tissues, are capable of transforming into cancer stem cells, depending on their genome integrity and external context. Disturbances of the genetic and epigenetic mechanisms that control the balance of self-renewal and differentiation in stem cells lead to functional impairments and malignant transformation into cancer stem cells [5,6,7,8].

Pluripotent and teratocarcinoma stem cells are well characterized and stable cell lines that represent unique models for studying the initiation, progression, and development of cancer. These paired models of normal and cancerous stem cells allow us to uncover the normal and transformed cell lineages and understand the mechanisms that regulate the signaling network during each step of multi-lineage differentiation and tumor progression [9].

Identifying critical nodes in the regulatory cascades initiated by TGFβ family factors and those that are shared by TGFβ family factors and other signaling pathways, as well as analyzing the rearrangements of the regulatory network during stem cell state changes, are key issues for understanding the fundamental mechanisms of both stem cell biology and cancer initiation and progression. This review summarizes recent advances in our understanding of the biological roles of TGFβ family factors in pluripotent stem cells and discusses how TGFβ family signaling pathways are involved in perturbations in the regulatory network of teratocarcinoma stem cells, which are the malignant counterparts of pluripotent stem cells.

## 2. Overview of TGFβ Family Signaling

### 2.1. Canonical and Non-Canonical TGFβ Family Signaling

**Figure 1:**
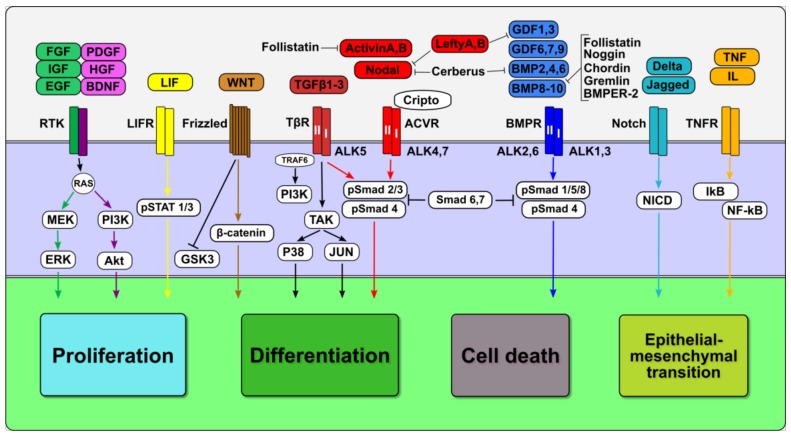
The transforming growth factor-β (TGFβ) family signaling pathways contribute to the context-dependent regulation of basic cellular processes though their interplay with the MEK/ERK, phosphatidylinositol 3′-kinase (PI3K)/ protein kinase B (Akt) (purple), WNT/GSK (brown), JAK/STAT (yellow), NOTCH (light blue), and NF-κB (orange) signaling pathways. TGFβ family ligands (TGFβ, Activins, Nodal, Lefties, bone morphogenic proteins (BMPs), and GDFs) bind to type I and type II transmembrane receptors and form an activated receptor complex, which phosphorylates R-Smads (Smad1-3,5,8) proteins. R-Smads-Smad4 complexes are translocated into the nucleus and directly or indirectly regulate the expression of numerous transcriptional factors that support proliferation, differentiation, the epithelial-mesenchymal transition, cell death, and survival. Smad6 and Smad7 antagonize signaling activation by binding to R-Smads and preventing their interaction with Smad4. The canonical TGFβ family signaling pathway contains two branches, TGFβ/ActivinA/Nodal/Smad2/3 (red) and BMP/GDF/Smad1/5/8 (blue), whereas non-canonical TGFβ cascades act through pathways activated by MAPKs (MKK/Jun/p38) (black). TGFβ family signaling pathways are modulated by various agonists and antagonists at different cellular levels.

TGFβ family factors (TGFβ, Activin, Nodal, Lefty, bone morphogenic proteins (BMP), and growth differentiation factors (GDF)) act through type I and II transmembrane serine-threonine receptors, which are associated with the kinases of the Activin receptor-like kinases (ALKs) [10] (Figure 1). There are seven type I receptors and five type II receptors, which are combined in receptor complexes for all the ligands of the TGFβ family. Activated receptor kinases phosphorylate Smad transducer proteins (Mad mothers against decapentaplegic homolog). Smad proteins are divided into three groups: receptor R-Smads (Smad1, 2, 3, 5, and 8), co-activator Co-Smad (Smad4), and inhibitor I-Smads (Smad6 and Smad7). Activated R-Smads cooperate with Smad4 and are translocated into the nucleus, where, in complexes with different transcription factors and co-factors, they activate or repress the expression of target genes. Inhibitory Smad6 and Smad7 antagonize signaling activation by binding to R-Smads and preventing their interaction with Smad4 [11]. However, since R-Smads-Smad4 transcription complexes have low affinities to DNA, they depend on cooperation with the transcriptional machinery, chromatin modifying co-activators p300 and CBP histone acetyl-transferases, SWI/SNF chromatin remodeling complexes and other transcription factors to bind to the regulatory sequences of enhancers and promoters of target genes [12]. Smads can interact and cooperate with numerous transcriptional factors, such as bHLH (basic helix-loop-helix), bZIP (basic leucine zipper), Foxhead, Homeodomain, Runx, and Zinc-finger protein families, as well as p53, β-catenin, Pax8, Sox9, Lef1/Tcf, and others [13,14].

There are two main canonical TGFβ family signaling pathway branches: TGFβ/ActivinA/Nodal/Smad2/3 and BMP/GDF/Smad1/5/8, which activate different target genes and often have opposite functional roles [10]. Non-canonical, non-Smad-mediated TGFβ cascades can activate TGFβ-activated kinases 1 (TAK-1) and act through pathways activated by different Mitogen-Activated Protein kinases, MAPKs (MKK/Jun/p38), phosphatidylinositol 3′-kinase (PI3K), protein kinase B (Akt), or Rho-like GTPases, as well as NF-kB, Notch, and Hippo pathways [15,16,17,18,19]. Furthermore, Smad- and non-Smad-mediated signaling branches can cooperate via TGFβ ligand-receptor-initiated interaction between Smad2/3 and NOX, p53, c-Src, YAP/TAZ, and other proteins [20,21,22].

TGFβ family signaling pathways are modulated by various agonists and antagonists at different cellular levels [23]. Extracellular soluble factors bind directly to TGFβ family factors and regulate their interactions with receptors, thus influencing general signaling outcomes. Among them, follistatins bind Activin, BMP-2, BMP-4, BMP-7, and GDF-8/Myostatin and neutralize signaling activity through the formation of different complexes that prevent the ligand-receptor association [24,25,26,27,28]. BMP signaling is inhibited by several antagonists, such as Noggin [29], Chordin [30], Twisted gastrulation [31], Cerberus [32], Gremlin 1 and 2 [33], and Sclerostin [34], using ligand-receptor interaction blocking mechanisms.

At the same time, BMPER-2 can act as both an activator and inhibitor of BMP signaling, depending on its interactions with extracellular matrix proteins [35,36,37]. Moreover, the activity of TGFβ family signaling pathways significantly depends on the interaction of the ligands with the proteoglycans and extracellular matrix proteins, which affect protein processing, diffusion and activity [38,39,40].

Several TGFβ family members can modulate the activity of the different branches of TGFb family signaling pathways. Lefty 1 and 2 antagonize Nodal, GDF-1, and GDF-3, but not Activin or TGFβ signals, by preventing receptor interactions, and GDF-3 and BMP-3 inhibit the signaling of BMP and Activin but not Nodal [23]. Importantly, the antagonists and agonists are implicated in feedback control of TGFβ family signaling pathways by regulating the expression of the TGFβ family ligands and vice versa; TGFβ family factors regulate the expression of their regulators.

TGFβ factors also contribute to indirect regulation of the expression of a number of master transcriptional factors. Thus, TGFβ directly and indirectly regulates C-myc expression and function through transcriptional repression by Smad3 and through derepression of cyclin D-dependent kinases (CDK), and p15^ink4B^ by reducing MYC-MIZ-1 interaction in its promoter, thereby enhancing anti-proliferative effects. In addition, TGFβ indirectly inhibits the expression of E-cadherin and Inhibitor of differentiation-2 (ID2) by inducing the expression of their repressors: a zinc-finger protein SIP-1 and activating transcriptional factor 3 (ATF3), respectively [1].

### 2.2. Crosstalk Between TGFβ Family and Other Signaling Pathways

TGFβ family signaling pathways are integrated in the cell regulatory network through synergistic and antagonistic interactions with other signaling pathways. The cross-talk between TGFβ family signaling branches and the MEK/ERK, PI3K/AKT, WNT/GSK, JAK/STAT, and NF-κB signaling pathways define the ultimate cell responses in cell-specific context [17,19].

The interplay between the TGFβ family and different MAPK pathways (MEK/ERK, JNK, and p38) includes the cooperation of both canonical and non-canonical cascades. MAPK pathways are activated through receptor tyrosine kinases (RTK) and a small membrane-bound GTPase, RAS, which transduces the signal to serine-threonine kinase, RAF, and the extracellular signal-regulated kinases ERK1/2 [41]. The ERKs activate the expression of a number of crucial transcription factors that regulate proliferation, apoptosis and migration. Many of these regulators, such as C-myc, p15^ink4b^, p21^Cip1^, and Id1-3, are also components and direct and indirect targets of TGFβ/BMP/Smads signaling [42,43,44,45]. In general, the RAS/ERK pathway antagonizes TGFβ-induced cell cycle arrest and apoptosis but also promotes the epithelial-mesenchymal transition [17]. ERK1/2, JNK, and p38 kinases can phosphorylate the linker regions of Smad2/3, Smad1/5, Smad4, and Smad7, block Smad nuclear translocation, and regulate the transcription of Smad7 [46,47,48,49,50]. Moreover, TGFβ family factors and MAPKs can reciprocally regulate the activity of each other’s signaling cascades via non-Smad TGFβ signaling and other mechanisms.

The PI3K/Akt and TGFβ family signaling pathways can antagonize each other to support the balance of cell growth, death and differentiation, but can also cooperate to promote cell survival. The PI3K/Akt signaling pathway, which is also activated through the RTK/RAS cascade, can promote cell growth and survival or facilitate TGFβ-induced cell cycle arrest and apoptosis, depending on physiological context. Akt can physically interact with Smad3, but not Smad2, and prevent TGFβ/Smad3-induced apoptosis [51,52,53]. TGFβ/Activin-induced up-regulation of the expression of SHIP phosphatase (Src homology 2 (SH2) domain-containing 5’ inositol phosphatase) results in inhibition of Akt/PKB (protein kinase B) and stimulation of apoptosis [54]. The PI3K/Akt signaling pathway modulates TGFβ-mediated cytostatic effects through inhibition of FoxO transcriptional factors, which form complexes with Smads and activate p21^Cip1^ expression [55]. In contrast, TGFβ can promote cell proliferation and survival via PI3K/Akt signaling and the p38 kinase cascade, independently of Smad2/3 in myofibroblasts, lung mesenchymal cells and hippocampal neurons [56,57,58]. TGF-β can activate PI3K and Akt through two Smad-independent pathways: by a direct interaction between PI3K, E3 ubiquitin ligase tumor necrosis factor receptor-associated factor 6 (TRAF6), and the TGF-β type I receptor or by the TGF-β type I receptor-independent activation of TRAF6, which polyubiquitinates the PI3K regulatory subunit p85α and facilitates the formation of a complex between the TGF-β type I receptor and p85α in the presence of SMAD7 [59,60].

The TGFβ/BMP and WNT/β-catenin signaling pathways’ crosstalk in the nucleus or cytoplasm is also essential for the regulation of the balance of cellular proliferation, differentiation, death and migration. The Smad-β-catenin-Lef1/Tcf protein complex, which forms in the nucleus during the interaction of the TGFβ/BMP and WNT cascades, is able to synergistically regulate the expression of target genes, such as *Msx1*, *Msx2*, *Emx2*, and *gastrin* [61,62,63]. The antagonistic BMP/WNT crosstalk influences Id1 expression and myoblast differentiation ability [64], and WNT-dependent maintenance/differentiation of the intestinal stem cells through BMP signaling modulation [65]. In addition, TGF-β/BMP and WNT cascades reciprocally regulate the expression of their ligands and antagonists. Thus, Wnt-8c/β-catenin signaling can regulate the expression of Nodal during left-right determination in chick embryos [66], whereas BMP-2 down-regulates Wnt-7a by activating p38 protein kinase in chicken embryonic mesenchymal cells [67]. The canonical Wnt/ β-catenin/Tcf signaling pathway directly regulates the expression of Cripto-1, which is a Nodal co-receptor [68]. Furthermore, Wnt signaling inhibits GSK-3β and thereby prevents phosphorylation in Smad protein linkers and stabilizes Smad proteins [69,70]. Direct physical interactions between Smad proteins and Wnt pathway components can also modulate the activity of each other. The interaction of Axin and Smad3 results in the phosphorylation of Smad3 by the TGFβ type I receptor kinase and enhanced transcriptional activation of Smad3 targets [71]. Through regulation of the interactions between Axin, GSC-3β, CKIε, and Smad3 proteins, TGFβ can induce nuclear co-translocation of β-catenin and Smad3 during the proliferation of human mesenchymal stem cells [72].

The crosstalk between the TGFβ/BMP and Notch signaling pathways varies depending on the cell context and the activity of other signaling pathways [73]. The TGFβ/Smad3 cascade can induce the expression of the Notch ligand, Jagged1, and the Notch target, Hey1, during the epithelial-to-mesenchymal transition [74]. Treating human kidney epithelial cells with TGFβ1 increased Jagged1 and Hes1 mRNA and stimulated the expression of a subset of TGFβ1-responsive genes that are involved in the epithelial-to-mesenchymal transition regulation [75]. Similarly, BMP2/4 can enhance Notch signaling and stimulate transcription of Notch target genes, Hes-1, Hes-5, Hey-1, and Hesr-1, and thereby suppress the differentiation of myoblasts, osteoblasts and neuroepithelial precursors [76,77,78]. Smad3, Smad1 and Smad5 proteins can directly interact with the Notch intracellular domain (NICD), and this complex is recruited to the promoters of key Notch target genes to synergize or antagonize the effects of both signalings [77,79,80,81]. A positive reciprocal regulatory feedback loop between Notch and TGFβ maintains prostate basal stem cells by upregulating TGFβ signaling components, including TgfβR1 [82].

TGFβ can activate NF-kB signaling, which also can mediate the transcription of both TGFβ and NF-kB target genes [83,84]. Activation of NF-kB by TGFβ/Smad-dependent mechanisms can be provided by direct protein-to-protein interactions between Smad3 and NF-kB or its activator IKKa [83,85,86]. TGFβ can also cross-talk with JAK-STAT signaling through the direct binding of Smad3 with STAT3 [87] or indirectly through interferon-γ/JAK/STAT1-mediated enhancement of Smad7 expression, which inhibits the phosphorylation of Smad3 [88].

### 2.3. Context-Dependent Activity and Roles of TGFβ Family Signaling

TGFβ family factors induce diverse cellular responses that depend on the cell type and physiological status. These context-dependent effects are governed by the complex multi-level regulation of TGFβ family signaling pathway components and interactions with other signaling pathways. Therefore, the outcomes of TGFβ family signaling-based regulation of proliferation, apoptosis, differentiation and migration vary significantly in different cells (Figure 1).

Inhibition of the cell growth in response to TGFβ in various cell types is associated with Smad3-mediated mechanisms that activate the expression of the CDK inhibitors, p15^ink4b^ and p21^Cip1^, as well as repressing the growth-stimulating transcription factors C-myc and Id1-3 [89]. An additional mechanism of TGFβ-induced cell proliferation arrest is associated with the repression of the expression or phosphorylation of the CDK tyrosine phosphatase Cdc25A [90]. On the other side, TGFβ can also stimulate proliferation in several mesenchymal cell types through Smad-independent mechanisms [91]. However, the growth- stimulating effects of TGFβ may be the result of crosstalk with the MAPK, PI3K/Akt, and Wnt/β-catenin/ GSC-3β signaling pathways [17,19,92].

TGFβ family factors affect cell death and survival by modulating the expression of both anti-apoptotic and pro-apoptotic genes, such as *Bcl-2* and *Bcl-X1* or *Bad* and *Bax* [93,94,95,96,97,98], as well as through a cooperation with the PI3K/Akt and NF-kB signaling pathways. The mechanisms of TGFβ-induced apoptosis may be specific to different cell types and involve the activation of expression of SHIP phosphatase [54], DAP kinase [99], growth arrest and DNA damage inducible protein (GADD45β) [100], connective tissue growth factor (CTGF) [94] and programmed cell death protein (PDCD4) [101].

TGFβ family factors are widely involved in the regulation of differentiation and morphogenesis during development and histogenesis. TGFβ family factors are able to initiate or inhibit the differentiation of certain cell types by regulating the expression of lineage-specific genes. Different branches of the TGFβ family signaling pathways may also have various effects in the same cell type during differentiation [102,103]. Moreover, different levels of TGFβ family factors define cell fate during embryonic lineage specification: low Bmp4 signaling directs ectodermal differentiation, whereas high Bmp4 levels, like Nodal/Smad2/3 signaling, results in mesoderm or endoderm specification [104,105,106,107]. The TGFβ/Activin/Nodal/Smad2/3 signaling pathway is indispensable for the maintenance of plupipotent stem cells but also can stimulate mesendodermal differentiation by inducing the expression of the homeobox genes, *Goosecoid (Gsc)* and *Mix-like homeodomain protein 1 (Mixl1)* [108,109,110,111]. At the same time, TGFβ inhibits the differentiation of skeletal myoblasts and osteoblasts through the interaction of Smad3 with MyoD and MEF2, as well as the interaction of Smad3 with Runx2 [41,112,113].

TGFβ signaling plays a key role in the epithelial-mesenchymal transition (EMT) during development and cancer metastasis [114]. TGFβ can induce the expression of negative regulators of E-cadherin expression, such as SIP1 and Snail, or through the repression of Id gene expression [115,116,117,118]. To induce EMT, TGFβ also cooperates with the MEK/ERK [119], p38 [120], NF-kB [121], and Notch [74] signaling pathways and their components, RAS and RhoA [15,122]. In response to TGFβ stimulation, both Smad7 and p38 regulate the expression of adenomatous polyposis coli (APC), which is involved in microtubule organization and prostate cancer cell migration [123]. Activation of the TGFβ-TRAF6-p38 pathway promotes the expression and activation of c-Jun, which can bind to the Snail gene promoter [124,125]. Additionally, TGFβ via TRAF6 promotes the proteolytic cleavage of TβRI in cancer cells, resulting in the liberation and nuclear translocation of its intracellular domain, subsequent association with the transcription regulator p300 and activation of Snail and MMP2 expression [126].

Deregulation of TGFβ/BMP signaling pathways during the progression of various types of cancer, resulting from genetic and epigenetic changes in the genes of the components of TGFβ family signaling pathways or their repressors, leads to changes in the intensity and duration of signals and to consistent reorganization of the cell regulatory signaling network. These changes are associated with decreased anti-proliferative and pro-differentiation responses to TGFβ/BMP signals in cancer cells [92,127,128]. The mechanisms that contribute to the deregulation of TGFβ family signaling pathways include mutations, promoter methylation and protein modifications of such regulators as phosphatase and tensin homologue (PTEN) [129,130], bone morphogenic protein 7 (BMP7) [131,132], SMAD7 [133], bone morphogenetic protein and activin membrane-bound Inhibitor (BAMBI) [20,134,135], Sloan-Kettering Institute proto-oncogene/Ski related novel gene (Ski/SnoN) [136], and Klotho proteins [137], which bind different growth factor receptors.

## 3. TGFβ Family Signaling Pathways in Regulation of Pluripotency, Self-Renewal, and Differentiation

### 3.1. TGFβ Family in Signaling Networks of Naїve and Primed Pluripotent Stem Cells

**Figure 2:**
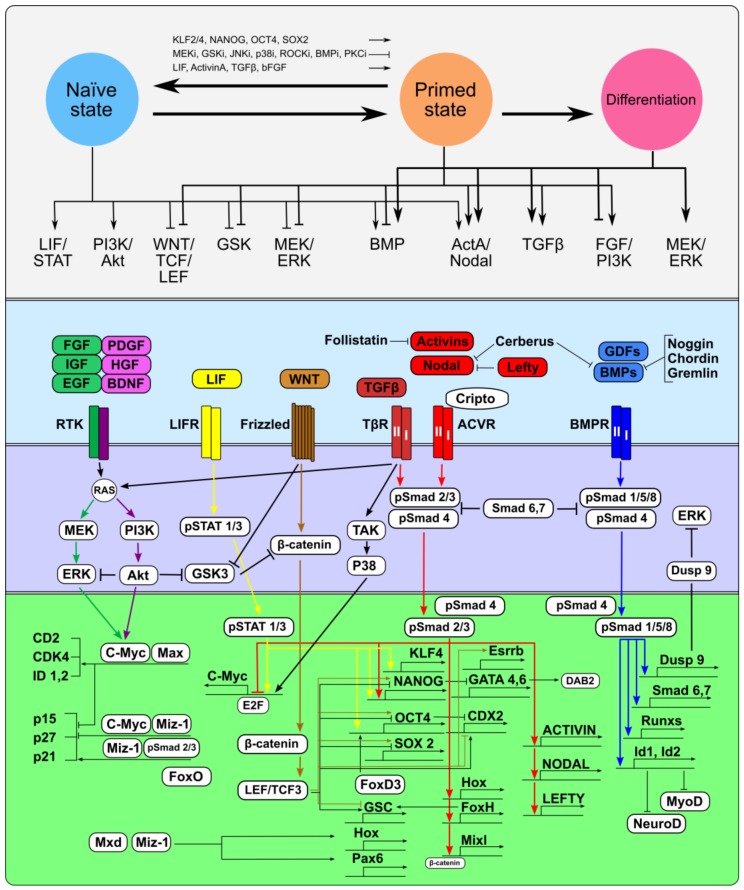
Signaling and gene regulatory networks in pluripotent (naїve and primed) and teratocarcinoma stem cells. Schematic representation of the TGF-β family, Leukemia inhibiting factor (LIF)/JAK/STAT, MEK/ERK, PI3K/AKT, and WNT/β-catenin/GSK3 signaling pathways and their targets that are involved in the regulation of self-renewal and differentiation of pluripotent and teratocarcinoma stem cells. The LIF/JAK/STAT3 pathway (yellow) activates core pluripotency factors (Oct4, Nanog, and Sox2) and Klf4 and promotes the naїve pluripotent state. The BMP contributes to the self-renewal of naїve pluripotent cells by inducing Id1-3 expression. Suppression of MEK and GSK3β with chemical inhibitors, together with LIF supplementation, facilitates the stabilization of naїve pluripotency and blocks differentiation stimuli. The reversal primed-to-naїve state conversion is achieved through the activation of ectopic expression of OCT4, SOX2, NANOG, KLF4, and KLF2 or exposure to various combinations of small molecule inhibitors and growth factors. The RTK/ PI3K/AKT signaling pathway (purple) supports the self-renewal of both the naїve and primed pluripotent states, whereas the receptor tyrosine kinases (RTK)/MEK/ERK signaling pathway (green) facilitates the transition from the naїve to the primed state. WNT signaling (brown) blocks GSK3 activity and stabilizes β-catenin, which reduces TCF3-mediated repression of pluripotency-specific genes. The bFGF/PI3K/Akt and TGFβ/ActivinA/Nodal signaling pathways stimulate the self-renewal of primed pluripotent stem cells. The TGFβ/Activin/Nodal/Smad2/3 (red) and BMP/GDF/Smad1/5/8 (blue) signaling branches are involved in the maintenance of the naїve and primed pluripotent states and in differentiation into different embryonic and extraembryonic lineages. Crosstalk between the TGFβ family and other signaling pathways and feedback within the TGFβ family signaling branches provide a dynamic equilibrium in the signal network of pluripotent stem cells, whereas TGFβ family signaling pathway rearrangements impair the differentiation of teratocarcinoma stem cells.

Pluripotent stem cells—embryonic stem (ESCs), embryonic germ (EGCs), epiblast stem (mEpiSCs) and induced pluripotent stem cells (iPSCs)—can be maintained indefinitely in a metastable and dynamic undifferentiated state in vitro by modulating the interplay of different signaling pathways. Undifferentiated pluripotent stem cells can exist in naїve and primed states, which represent the pre- and post-implantation epiblast developmental stages, respectively [138]. However, the self-renewal of pluripotent cells, which transiently exist in embryos, is limited, in contrast to pluripotent stem cell lines maintained in vitro. The main characteristics of pluripotent stem cells in both states, such as the expression of core transcription factors Oct4, Nanog, and Sox2, cell cycle structures and multilineage differentiation potential, are significantly similar, whereas other characteristics, such as germ line transmission in chimeric embryos, X-chromosome inactivation and single-cell viability/clonogenicity substantially differ [139,140]. For the maintenance of pluripotent states and self-renewal, intrinsic core transcription factors Nanog, Oct4, Sox2, Esrrb, and Klf4 form an autoregulatory circuit, which is supported by extrinsic signaling factors [141,142,143]. However, naїve and primed pluripotent stem cells are maintained in different in vitro systems supporting adequate configurations of their gene regulatory network that simultaneously ensure their identity, stimulate self-renewal and inhibit differentiation. At the same time, pluripotent stem cells can differentiate into various embryonic and extraembryonic cell lineages when intrinsic signals from the extraembryonic microenvironment or from the culture medium induce changes in the extrinsic signaling network by increasing the pool of anti-proliferative and pro-differentiation signals (Figure 2).

The self-renewal of naїve pluripotent stem cells, represented with mouse ESCs (mESCs) as the in vitro prototype, requires the activity of the Leukemia inhibiting factor (LIF)/Jak/Stat3 signaling pathway in cooperation with the TGFβ/BMP and PI3K/Akt signaling pathways [144,145,146,147,148,149,150,151,152], whereas Fibroblast growth factor 4 (Fgf4) stimulates Erk signaling, thus contributing to the exit from the self-renewal stage and the onset of lineage commitment [153]. In primed pluripotent stem cells, represented by mouse epiblast stem cells (mEpiSCs) and human ESCs (hESCs), self-renewal is ensured by the FGF2/PI3K/Akt and TGFβ/ActivinA/Nodal signaling pathways [154,155], whereas the LIF/Jak/Stat3 signaling pathway is dispensable [156]. FGF2, as well as insulin-like growth factor-1 (IGF1), epidermal growth factor (EGF), platelet-derived growth factor (PDGF), and other growth factors, can activate the PI3K/Akt signaling pathway and support the self-renewal and viability of hESCs [155,157,158,159]. However, FGF2 alone without cooperation from TGF/ActivinA/Nodal is not sufficient to stimulate the self-renewal of primed hESCs [155,160,161], although the combination of ActivinA and FGF2 may also activate MEK/ERK signaling and induce hESC differentiation [161].

For both naїve and primed pluripotent stem cells, the MEK/ERK signaling pathways are involve in supporting the proliferation of differentiating cell descendants, but not in the self-renewal of pluripotent stem cells. Inhibition of MEK1/2 and ERK1/2 by chemical inhibitors enhances the proliferation and prevents differentiation of ESCs [161,162,163]. However, knockout of Erk1 in mESCs leads to telomere shortening and genomic instability, as well as reduced cell proliferation, cell cycle arrest, and increased apoptosis, whereas constitutively active Mek1, but not Erk1 or Erk2, suppresses *Nanog* expression [164]. In addition, data on the role of the MEK/ERK signaling pathway in hESCs are also contradictory: inhibition of ERK1/2 can facilitate the undifferentiated hESC phenotype, blocking their differentiation into mesodermal and neuroectodermal cells [161], and it can induce hESC differentiation and cell death [165,166].

In both phases of pluripotency, the threshold levels of activity of the serine/threonine protein kinase Gsk3β, implicated in the PI3K/Akt, MEK/ERK and Wnt/β-catenin signaling pathways, result in pleiotropic biological effects. In naїve mESCs, PI3K/Akt suppresses Gsk3β, which antagonizes self-renewal by targeting Myc and Nanog; in primed hESCs, Gsk3β is also involved in suppression of canonical Wnt signaling [167,168]. Wnt/β-catenin signaling supports self-renewal and inhibits the transition of naїve mESCs and induced naїve hESCs to the primed state [169].

TGFβ family signaling is critical for supporting the identity and self-renewal of both naїve and primed pluripotent stem cells, while the contribution of the underlying mechanisms of the ActivinA/Nodal and BMP signaling branches might differ. Noteworthy is that, in both naїve and primed pluripotent stem cells, ActivinA/Nodal/Smad2/3 signaling activates the expression of the key pluripotency transcriptional factor Nanog [170,171]. Additionally, Smad3 and Smad2 co-occupy the genome with Oct4, Nanog, and Sox2, including the promoters of these genes themselves [103,172]. Inhibiting Smad2 and Smad3 phosphorylation with aSB431542 inhibitor, and through Smad7, Lefty, and Follistatin, results in the down-regulation of Oct4 and Nanog and decreased proliferation of both cell types [155,170,173,174,175].

The activity of the BMP/Smad1/5/8 signaling branch contributes to the self-renewal of naїve mESCs by inducing the expression of Id1 and Id3, which inhibit the expression of the transcription factors MyoD and NeuroD and differentiation into the mesodermal and neuronal lineages, respectively [176]. Smad1 with Oct4, Nanog, Sox2, and Stat3 co-occupies the same sites in the mESC genome [142]; and Smad1 can physically interact with Nanog and block BMP-induced mesoderm differentiation [177]. In addition, BMP/Smad1/5 signaling activates the expression of the dual-specificity phosphatase Dusp9, which inhibits Mek/Erk signaling, and together with LIF reinforces the self-renewal of mESCs [178]. However, in contrast to Activin/Nodal, inhibition of BMP signaling by Smad6 did not have an effect on mESC proliferation, indicating that this pathway may be dispensable for self-renewal [174]. At the same time, BMP represses self-renewal and promotes the differentiation of primed hESCs [111,155,160,179,180,181,182].

Furthermore, differences in the regulation of TGFβ family signaling pathways in naїve and primed pluripotent stem cells may be associated with the endogenous expression levels of these factors. A comparative quantitative analysis of the endogenous gene expression of TGFβ family factors identified significant differences between the naїve and primed pluripotent stem cells grown in serum-free medium [183]. Thus, the highest expression levels were detected for *Lefty1* in the mESCs and for *TGFβ1* and *BMP4* in hESCs; additionally, *NODAL*, *TGFβ1*, *BMP4,* and *GDF3* expression was higher, while *ACTIVINA* and *LEFTY1* expression was lower, in hESCs than in mESCs. However, with the onset of spontaneous differentiation, the expression profiles of the TGFβ family factors in hESCs and mESCs became similar: high levels of *BMP4* and *TGFβ1* and significantly decreased expression levels of *ACTIVINA/ActivinA*, *NODAL/Nodal*, *LEFTY1/Lefty1*, and *GDF3/Gdf3.* Therefore, the pro-differentiation effects of BMP signaling in hESCs can be governed by significantly higher endogenous BMP4 expression compared to mESCs. Given that plenty of potential targets are activated by the signaling pathways of TGFβ family members, the time-dependent concentrations of each factor and interactions between their targets contribute to the final cellular status/fate.

### 3.2. Signaling Pathway Rearrangements during Interconversion Between Naїve and Primed Pluripotent States

During early development pluripotent cells transit from the naїve to primed phase and then move toward the exit from the pluripotent state and toward the onset of differentiation. The natural fluctuations in the expression levels of key transcription factors at the single-cell level can induce rearrangement of the entire signal-regulatory network and trigger a transition to the next state [184,185,186]. To block this natural trajectory of the embryonic cells and maintain a metastable pluripotent state in vitro, it is necessary to apply exogenous factors that repress the expression of intrinsic factors, which induce the exit from self-renewal and toward the activation of differentiation programs. Therefore, modulation of the LIF/JAK/STAT3, MEK/ERK, WNT/β-catenin, and TGFβ family signaling pathways is important to maintain pluripotent states and to commit the cell to particular fates.

Suppression of MEK and GSK3β with chemical inhibitors (2i: PD0325901 and CHIR99021, respectively), together with LIF supplementation (2iL), stabilize naїve pluripotency and block differentiation stimuli in mESCs [138,163]. LIF/JAK/STAT3 signaling activates several pluripotency-promoting targets, such as *Klf4*, *Gbx2*, *c-Myc*, and *Tcfp2l1* [152,187,188]. In contrast, the FGF/MEK/ERK pathway can drive the transition from the naїve state to primed state and lineage commitment, in particular, by enhancing proteasomal degradation of the pluripotency-promoting factor Klf2 [189]. Therefore, inhibition of FGF/MEK/ERK signaling, as well as genetic knockout of *Fgf4* and *Erk2*, in mESCs leads to significant disturbances in differentiation and retention of pluripotency factor expression [153]. The effects of GSK3β inhibition on mESC self-renewal are mediated through the stabilization of β-catenin, which translocates into the nucleus and enhances the expression of pluripotency factors via disruption of Tcf3-mediated repression of the Oct4/Sox2/Nanog expression circuit [190]. The critical target downstream of GSK3/TCF3 inhibition is ESRRB, which can directly bind to Nanog and maintain self-renewal and pluripotency [191,192]. In this way, the MEK/ERK/Klf2 and GSK3/TCF3/ESRRB pathways interact to capture the naїve pluripotent state. Stabilization of naїve pluripotency under 2iL conditions can occur with more homogeneous expression of pluripotency factors since the more effective suppression of stimuli inducing the expression of lineage differentiation markers [139,184,193]. Apparently, in naїve pluripotent cells, the endogenous expression of TGFβ family factors and the activity of their signaling branches are directed to balance the signals, which can regulate the expression of pluripotency and lineage-specific factors.

When modeling interconversions of pluripotency phases in vitro, naїve mESCs transit to primed mEpiSCs after changing conventional culture conditions by supplementing the media with Activin A and bFGF [194]. At the same time, the reversal primed-to-naїve state conversion was impossible when the mEpiSCs were transferred to naїve state-promoting culture conditions with 2iL, but it occurred in mEpiSCs that overexpressed *Klf4*, *Klf2*, and *Nanog* [150,194,195,196] The transition of primed hESCs/hiPSCs to naїve pluripotent cells was achieved by using strategies that involved genetic modification with ectopic expression of *OCT4*, *SOX2*, *NANOG*, *KLF4*, and *KLF2* [17,197,198,199] or exposure to various combinations of small molecule inhibitors and growth factors [3,139,140]. During the reverse transition, hESCs restore LIF responsiveness and require the presence of 2i (MEKi and GSK3i), which promotes the selection and stabilization of cells in the naїve state [197,198,199,200,201,202,203,204,205]. Simultaneously, hESCs remain dependent on bFGF, Activin A or TGFβ and need exposure to additional inhibitors, such as JNKi (SP600125), p38i (SB203580), ROCKi (Y-27632), forskolin, BMPi (dorsomorphin), protein kinase C inhibitor Gö6983, and histone deacetylase inhibitors (sodium butyrate and suberoylanilide hydroxamic acid), to maintain pluripotent naїve state [198,199,200,201,202,203].

Noteworthy is that, when reprogramming the somatic cells of different mammals into iPSCs, the resultant cultures are stabilized in those phases of pluripotency that are characteristic of their ESCs [140]. Thus, miPSCs are derived and maintained in LIF-containing media [206], whereas conventional media for human and monkey iPSCs contains Activin A and bFGF [207,208,209]. On the other hand, in vitro conversion of mouse and human primordial germ cells to EGCs, whose characteristics are more similar to those of naїve ESCs, does not require genetic modification or ActivinA or TGFβ in the medium, but does require LIF and bFGF supplements in the medium [210,211,212,213,214,215].

Thus, the stabilization of the naїve state requires the weakening of TGFβ family signaling pathways to balance pro-differentiation stimuli, although their activity is necessary to maintain the expression of core pluripotency transcriptional factors. The reverse transition from the primed to the naїve phase of pluripotency occurs under conditions where the MEK/ERK signaling pathway and GSK3β activity are weakened, LIF/STAT3/KLF2/4 signaling is enhanced, and the cells remain partially dependent on bFGF and Activin A/TGFβ.

### 3.3. TGFβ Family Signaling during the Onset of Pluripotent Stem Cell Differentiation

The gradually increasing heterogeneity of the expression of internal and external regulators during the transition from the naїve to the primed state of pluripotency and to the subsequent onset of differentiation is a necessary condition for the irreversibility of differentiation and development. Pluripotent stem cells are capable of initiating multilineage differentiation into somatic and germ cells, as well as extraembryonic tissues, in vivo and in vitro due to modulation of the spatiotemporal expression patterns of the TGFβ family factors. TGFβ family factors contribute to the maintenance of identity and self-renewal of undifferentiated pluripotent stem cells, but they become lineage-promoting drivers when the cells exit from the pluripotent state and enter differentiation (Figure 2).

Balancing self-renewal and differentiation in pluripotent stem cells, TGFβ family signaling pathways regulate cell cycle progression and initiate cell proliferation arrest. Cell cycle regulators, including cyclin D and CDK4 and CDK6, can directly affect Nodal/Activin/Smad2/3 signaling through the regulation of site-specific phosphorylation and nuclear translocation of the Smads2/3 [216,217]. Moreover, these regulative mechanisms are cell cycle-specific for the cell fate decision. In the early G1 phase, cyclin D and CDK4/6 are expressed at low levels, and therefore, Nodal/Activin/TGFβ signaling kinases phosphorylate the carboxyterminal region of the Smad2/3 proteins, which move to the nucleus and induce endoderm differentiation. In contrast, after entering the late G1 phase and up-regulating the expression and activity of cyclin D, CDK4, and CDK6, Smad2/3 proteins are phosphorylated in the linker regions and are not translocated into the nucleus; therefore, only neuroectodermal differentiation can be induced [217].

The Nodal/Activin/Smad2/3 signaling branch is critical for the activation of Nanog expression, and therefore, for the self-renewal and supporting of both naїve and primed pluripotency [170,171,218]. Inhibition of Activin/Nodal/Smad2/3 signaling by follistatin or SB431542 and by overexpression of Nodal antagonists Lefty or Cerberus, leads to neuroectoderm differentiation of hESCs [111,155]; whereas treatment with Activin and Nodal, when bFGF is depleted, results in mesendoderm differentiation [111,219]. Sustained activation of Activin signaling stimulates the differentiation of the primitive streak population: high and low levels induce definitive endoderm and mesoderm, respectively [220]. BMP/Smad1/5/8 signaling decreases the expression of pluripotency factors, NANOG and SOX2 [170,221], and, in cooperation with Activin/Nodal signaling, leads to expansion of the endodermal lineage. However, in the absence of Activin/Nodal and FGF signaling activity, BMP signaling promotes the differentiation of trophoblast and extraembryonic endoderm lineages [111,179,218]. Finally, the synergistic action of inhibitors for both branches of the TGFβ family signaling pathways, such as Noggin and SB431542, facilitate the induction of neuroectodermal lineages in hESCs and iPSCs [222].

During early differentiation, Smad 2/3 cooperate with new dominant transcriptional partners and activate the expression of quiescent lineage-specific master regulators that previously were in a so-called “poised” state [102,172,223]. Thus, Smad 2/3, along with Foxh1 and Eomes, co-occupy the genome sites for endoderm lineage regulators, or a Smad 2/3 associate with different combinations of transcription factors at previously activated gene enhancers [107,172,224,225]. In hESCs, Activin/Nodal/Smad2/3 signaling cooperates with NANOG, OCT4, and SOX2 to repress the expression of Smad-interacting protein 1 (SIP1), which inhibits mesendodermal and endodermal differentiation; however, SIP1 promotes neuroectodermal differentiation after Nodal/Activin signaling is reduced [226]. To switch mesodermal lineage genes from the “poised” to the active state, Smad2/3 form complexes with Trim33/TIF1γ that interact with H3K9me3 and H3K18ac on the promoters of *Gsc* and *Mixl1*. The Smad2/3-TRIM33-H3K9me3 complex displaces the chromatin-compacting factor HP1γ and thereby makes Activin/Nodal response elements accessible to Smad4-Smad2/3 for Pol II recruitment [102,223].

The multilevel interactions between the TGFβ signaling branches and PI3K/Akt, MEK/ERK and WNT/GSK3β/β-catenin signaling cascades play an important role in the formation of heterogeneity in pluripotent stem cell populations entering differentiation. Reduced PI3K/Akt activity weakens the suppression of the MEK/ERK and Wnt signaling pathways and redirects ActivinA/Smad2/3 signaling from a pro-self-renewal to a pro-differentiation function [128,168]. In light of these data, Nodal/Activin /Smad2/3 signaling is a crucial switch for regulating the balance between cell states.

## 4. Imbalance of TGFβ/BMP Signaling Pathways in Teratocarcinoma Stem Cells

### 4.1. Aberrant Characteristics and Cell States of Malignant Embryonal Carcinoma (Teratocarcinoma) Cell Lines

The malignant counterparts of the pluripotent stem cells—the embryonal carcinoma (teratocarcinoma) cell lines (ECCs)—have a germ cell /embryonic stem cell origin because they were isolated from mouse and human gonadal or extragonadal teratocarcinomas [227,228,229,230,231,232,233,234,235,236,237,238]. Importantly, the ECC lines exhibit karyotypic and genetic alterations in their genomes, which cause their cancer transformation and progression [232,233,238,239,240,241,242]. Genetic instability in mouse and human ECCs is associated with gene mutations (amplifications and point mutations) and enhanced expression of the *c-Ki-ras-2*, *N-ras*, and *N-myc* oncogenes [240,241,242,243,244]. The ECC lines exhibit multipotentiality, but have restricted differentiation potential in vitro and in vivo and during development in chimaera embryos, whereas the proliferative potentials of the undifferentiated ESCs and ECCs are similar [245,246,247,248,249,250,251,252]. Moreover, several ECC lines are considered nullipotent because they have completely lost the ability to differentiate spontaneously [232,238,252,253].

The ECC lines share many characteristics with pluripotent stem cells (ESCs, EGCs, and iPSCs) maintained in vitro: expression of core transcriptional factors (Oct4, Nanog, and Sox2) and cell surface antigens (SSEA3, SSEA4, TRA-1–60, and TRA-1-81), and cell cycle similarity [9,254]. However, the expression of numerous intrinsic and extrinsic regulators significantly differs between ECCs and pluripotent stem cells. A comparison of the transcriptional profiles of the 2102Ep and NTERA-2 hECC lines and the BG01, BG01V, and BG03 hESC lines showed their significant similarity, although the microRNA profiles for these lines were slightly different [255]. Transcriptome analysis of NTERA-2 hECCs and chHES-20 hESCs revealed upregulated expression of *POU5F1*, *NANOG*, *LDB2*, *GABRB3*, *FGF4*, *FGF13*, *DNMT3B*, *LDB2*, and *CD9* genes and decreased levels of lineage marker genes in hECCs, compared to the hESCs [256]. The gene expression data suggested that the Wnt and Notch signaling pathways may be key contributors to the carcinogenesis of NTERA-2 cells [256]. Quantitative proteomic analysis of H1 (WA01) hESCs and NTERA-2 hECCs identified nearly 200 differentially expressed proteins, among which were the early developmental regulators UTF1, DNMT3B, IFITM1, GDF3, CD99-antigen, CRABP2, and DPPA4, as well as the cancer-associated proteins, MAGEA4, HSPB1, MAP3K1, NFKBIL2, and S100A4 [257]. In addition to differences in the expression levels of pluripotency and lineage-specific markers, differences in the expression patterns of genes of the Melanoma antigen (MAGE) family were also identified between mouse and human ESCs and ECCs [258,259]. Comparative transcriptome studies of human normal and genetically abnormal pluripotent stem cell lines, hECC lines and samples of various germ cell tumors (teratocarcinomas, teratomas, seminomas, choriocarcinomas, and yolk sac tumors) revealed significant similarities between hESCs and hECCs compared to germ cell tumors [260,261,262]. All types of germ cell tumors and ECCs expressed high levels of 58 genes associated with genomic imprinting and the regulation of pluripotency (*NANOG*, *OCT4/POU5F1*, *GAL*, *DPPA4*, *NALP7*, etc.). At the same time, the analysis of normal hESCs and genetically altered (“adapted”) hESCs with genetic disorders did not reveal significant differences in their transcriptional profiles [263].

Comparative studies of mouse and human ECC lines have revealed variable characteristics and differences from normal and “adapted” pluripotent stem cells; however, how ECC states correspond to the naїve and primed states of normal pluripotent stem cells remains unclear. Given their germ cell/embryonic stem cell origin, ECC lines can be considered malignant counterparts of pluripotent stem cells, which partially or completely lose their ability to differentiate into embryonic somatic and germ cell lineages, although they retain proliferative potential comparable to that of normal cells. The imbalance of the proliferative and differentiation potentials in ECC lines is a result of the selection of genetically altered cells with high viability and growth rate and restricted differentiation capacities during cancer progression.

### 4.2. TGFβ Family Signaling Pathways Contribute to the Imbalance of Self-Renewal and Differentiation in Embryonal Carcinoma Cells

The deregulation of pro-mitogenic and pro-differentiation signal balance and intrinsic transcriptional networks impairs ECC differentiation (Figure 2). The self-renewal of both mouse and human ECCs does not require the activity of the LIF/STAT3 pathway, unlike naїve pluripotent stem cells, but depends on serum mitogenic factors, like primed pluripotent stem cells [264,265,266,267]. However, LIF can inhibit the retinoic acid (RA)-induced differentiation to extraembryonic endoderm lineages in OTF9 and P19 mECCs [264,268,269] or potentiate neural induction in P19 mECCs [270,271]. At the same time, growth factor-induced activity of PI3K/Akt and MAPK signalings is indispensable for supporting ECC self-renewal and viability [272,273], and these pathways cooperate with others and coordinate the phosphorylation of pluripotency and lineage-specific factors during differentiation [274,275].

Interestingly, unlike mESCs, F9 and P19 mECCs are not able to restore the naїve state when monolayer cultures are maintained in the naїve state-promoting culture conditions with 2iL. The ECC clusters in both lines became multilayer, like the colonies of mESC and mEGC, and the alkaline phosphatase activity significantly increased in the treated ECCs compared to the control cultures. However, no significant differences in the growth rates, cell distributions among cell cycle stages or gene expression were identified [276].

The activity of TGFβ family signaling pathways varies in different ECC lines, but these pathways are critical for supporting cellular identity and initiating differentiation [277]. The gene expression analysis of TGFβ family factors in pluripotent stem and teratocarcinoma cells identified lower expression of *ActivinA* in F9 and P19 mECCs than in mESCs and mEGCs, whereas the expression levels of *Nodal, Lefty1*, *TGFβ1*, *BMP4*, and *GDF3* were similar. In nullipotent PA-1 hECCs, *ACTIVINA*, *NODAL*, *LEFTY1*, *BMP4*, and *GDF3*, but not *TGFβ1*, were expressed at significantly lower levels than in ESM01 hESCs [278]. Moreover, different clones of PA-1 hECCs expressed high levels of Follistatin, which prevents the antiproliferative effect of exogenous ActivinA and maintains the rapid cell growth, whereas Activin overexpression decreases PA-1 cell proliferation, even in the presence of Follistatin [279,280]. Additionally, the expression and affinity of TGFβ receptors were found at low levels in undifferentiated F9 and PC-13 ECCs; therefore, there were low responses to these factors in the undifferentiated cells, but these responses increased after RA-induced differentiation [281]. In contrast, Activin receptors Acvr1b and Acvr2b are expressed in both undifferentiated and differentiated P19 and F9 mECCs and Tera-2 hECCs, whereas the expression of Activins and Inhibins greatly varied between these cells [267,282,283].

The BMP/Smad1/5/8 signaling branch contributes to the self-renewal and viability of PA-1 hECCs via stimulation of Id1/Id3 expression, whose inhibition by a peptide aptamer, Id1/3-PA7, induces apoptosis and cell-cycle arrest through increased expression level of cyclin-dependent kinase inhibitor the CDKN2A [284]. Similarly, inhibition of BMP/Smad1/5/8 signaling with dorsomorphin (DMH1) in PA-1 hECCs led to decreased growth via cell cycle arrest (Gordeeva, unpublished data). However, BMP/Smad1/5/8 signaling is dispensable for P19 mECC self-renewal, but plays a pivotal role in cardiogenic differentiation through cooperation with the MAPK/TAK1 pathway, which induces Csx/Nkx-2.5 and GATA-4 expression [285,286].

Despite significant differences between the levels of *ActivinA* and *Nanog* expression in ECCs and ESCs, a positive correlation was found between the expression levels of *ActivinA* and *Nanog*, but not *Oct4*, in both types of cell lines, indicating that similar mechanisms regulate *Nanog* expression via the Activin/Smad2/3 signaling branch [259,278]. Nanog stimulates Rex-1 expression, which is required to maintain undifferentiated F9 and P19 mECCs and downregulates the expression of primitive endoderm and parietal endoderm differentiation markers *Gata-6*, *Gata-4, Hnf1*, and *LamininB1* [287,288]. In undifferentiated F9 and P19 mECCs with low *Nanog* expression, enhanced expression of *Gata-4*, *Pax6*, and *Bry* was detected [259]. Similarly, NTERA2 hECCs, as well as some cell lines from human germ cell tumors, has aberrant *BRY* expression, and its expression decreased slightly after RA-stimulated neuronal differentiation but without differentiation into the mesodermal derivatives [289]. Therefore, aberrant expression of lineage-specific markers in undifferentiated ECCs may indicate the impaired regulation of their expression, but not initiation of cell differentiation.

Mutations in the *Ki-ras*, *N-ras*, and *N-myc* oncogenes and tumor suppressors that lead to changes in their expression levels play a pivotal role in the formation of an imbalance between the self-renewal and differentiation of ECCs [240,241,242,243,244]. Although some metastatic embryonal carcinomas showed mRNA overexpression without *c-Ki-ras2* gene amplification [242], sustained enhanced expression of Ras and Myc causes of hyperactivation of the RAS/MEK/ERK and PI3K/Akt signaling pathways and, accordingly, enhanced cell proliferation. Hyperactivation of these signaling pathways due to *N-ras* mutation can also lead to a significant reorganization of the signaling network by reducing the endogenous expression of *ACTIVINA*, *NODAL*, *LEFTY1*, *BMP4*, and *GDF3* and the activity of the corresponding signaling pathways [240,241,278,280].

Most ECC lines are not capable of spontaneous differentiation in monolayer cultures, but can be induced to differentiate with RA, hexamethylene bisacetamide (HMBA) and dimethyl sulfoxide (DMSO) or culturing in spheroids—embryoid bodies [235,290,291]. ECC differentiation is weakly induced by the TGFβ family factors alone. TGFβs or Activins could not induce the differentiation of P19, F9, and PA1 ECCs into embryonic lineages, but instead reduced cell proliferation or inhibited RA-induced differentiation [280,281,292]. At the same time, BMP7 inhibited proliferation and induced the differentiation of multipotent NTERA2 hECCs, whereas in nullipotent 2102Ep, 833KE and TERA-1 hECCs it elicited only a limited and partial response [293]. Interestingly, BMP2 stimulated the differentiation of GCT 27X-1 and NTera2/cloneD1 (NT2/D1) hECCs into different lineages—endodermal and non-neural ectodermal precursors, respectively [294,295]. However, the activity of the TGFβ family signaling pathways was significantly intensified in the course of the RA-, HMBA-, and DMSO-induced differentiation into different embryonic and extraembryonic cell lineages [282,283,285,286,292,296,297]. Noteworthy is that epigenetic changes that contribute to the deregulation of ECC differentiation—altered DNA methyltransferase (DNMT) expression/activity and the global methylation, and histone methylation and acetylation—can be regulated more efficiently by RA or 5-azacytidine than by TGFβ family factors [291,298,299,300,301,302,303,304].

**Figure 3:**
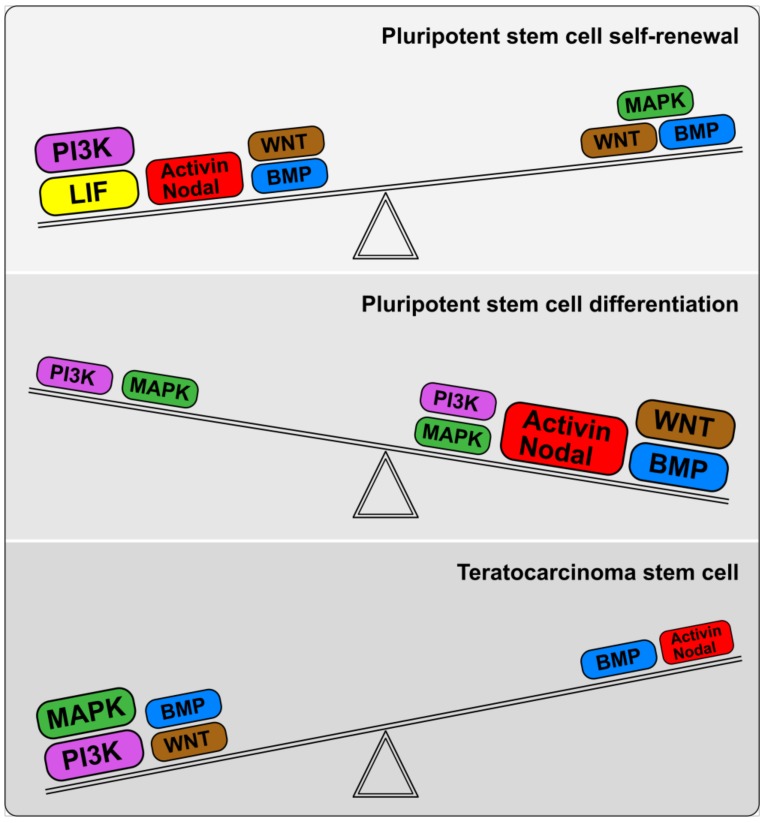
The balance of signaling pathways in the regulatory networks of pluripotent and teratocarcinoma stem cells. The LIF/JAK/STAT3, ActivinA/Nodal/Lefty/Smad2/3, PI3K/AKT, and BMP/Smad2/58 signaling pathways promote the maintenance of pluripotent stem cell identity and self-renewal, although the BMP/Smad1/5/8 signaling branch contributes to the regulation of pluripotent stem cell proliferation and differentiation. The MAPK pathway supports viability and induces differentiation. WNT signaling blocks GSK3 activity and stabilizes the expression of pluripotency-specific genes. The increased activity of the ActivinA/Nodal/TGFβ/Smad2/3 and BMP/Smad1/5/8 signaling pathways activates lineage-specific gene expression and thus induces different embryonic lineages. The activity of both the PI3K/AKT and MAPK signaling pathways is significantly attenuated during differentiation, but they contribute to the proliferation and viability of stem cell descendants. WNT/β-catenin signaling induces differentiation through the TCF3-mediated expression of lineage-specific genes. In teratocarcinoma stem cells, both the PI3K/AKT and MAPK signaling pathways are up-regulated, whereas ActivinA/Nodal/TGFβ/Smad2/3 signaling pathway activity is reduced. Reduced TGFβ family signaling pathway activity leads to various rearrangements in gene regulatory networks and impairs the differentiation potential of teratocarcinoma stem cells.

Thus, the stabilized signal-regulatory network configuration for each ECC line is based on the altered functions of mutant components and has a characteristic weak response to pro-differentiation signals, particularly TGFβ family factors (Figure 2 and Figure 3).

When compared with pluripotent stem cells, undifferentiated ECCs exist in the intermediate states that are not consistent with both pluripotency states: their self-renewal does not depend on either the LIF/STAT3 or ActivinA /Nodal pathways, but relies instead on both the PI3K/Akt and MAPK signaling pathways. Notably, the culture systems for the maintenance of both mouse and human ECCs are similar, unlike mouse and human ESCs. In addition, BMP/Smad1/5/8/Id1/3 can regulate cell cycle progression, apoptosis and early differentiation events similar to both naїve and primed pluripotent cells. Therefore, these fundamental differences between the gene regulatory networks of pluripotent and teratocarcinoma stem cells determine the different regulatory pathways for the onset of differentiation.

## 5. TGFβ Family Signaling Pathways in Regulation of Tumorigenicity of Pluripotent and Teratocarcinoma Stem Cells

Pluripotent stem cells and ECCs transplanted ectopically into adult animal tissue initiate rapidly growing tumors—teratomas and teratocarcinomas. ECCs’ limited differentiation and tumorigenicity are caused by their imbalanced signaling pathways. Although most ECC lines are only capable of partial differentiation, some ECC lines and sublines can completely differentiate in the course of long-term stimulation in vitro, and their descendants lose tumorigenicity after transplantation into recipients. Paradoxically, genetically normal pluripotent stem cells can give rise to teratomas that consist of the cell derivatives of different differentiation degrees. Teratoma growth initiation is also associated with the residual undifferentiated cells among in vitro differentiated pluripotent stem cell descendants. Why the residual undifferentiated ESCs retain among differentiated cells remains unclear but is a great challenge for the development of safe stem cell-based therapies.

The tumorigenicity of pluripotent stem cells is a consequence of the deregulation of the differentiation in ectopic tissue sites and autonomous regulation of cellular processes in the grafts. Moreover, the degree of cell differentiation in grafts that occurs after the transplantation of pluripotent stem cells is associated with chromosomal and gene abnormalities, as occurs in genetically abnormal teratocarcinoma cells [305,306,307,308,309]. Several studies have shown that the efficiency of experimental tumor growth after transplantation of both ESCs and ECCs depends on the immune status of the recipients and tissue site microenvironment [252,310,311,312,313]. However, the tumorigenicity and differentiation efficiency of naїve and primed pluripotent stem cells are also significantly different [252].

The differentiation patterns of ESCs and ECCs in the grafts are very similar to those of their embryoid bodies during spontaneous in vitro differentiation [267]. Nullipotent ECCs form tumors that consist mainly of undifferentiated cells, whereas multipotent ECC lines differentiate into multiple lineages [276]. In embryoid bodies and tumors formed by mESCs, mEGCs and F9 mECCs, similar gene expression profiles were revealed for both pluripotency and lineage markers, as well as for TGFβ family factors [267]. Interestingly, during the evolution of the F9 mECC line in vitro, the arising sublines exhibited different potentials for spontaneous and RA-induced differentiation. [314].

A detailed comparative study of RA-induced differentiation of mESCs and F9 mECCs revealed differences in the expression dynamics of TGFβ family factors and the loss of tumorigenicity in F9 mECC descendants after 10 days of RA exposure [297,315]. In the course of RA-induced differentiation, the expression levels of Activin A and BMP4 increased more sharply in the F9 mECCs than in mESCs, and these alterations contributed to the complete ECC differentiation into extraembryonic endoderm cells. Therefore, stimulation of the Activin/Nodal and BMP signaling cascades or inhibition of the MEK/ERK and PI3K/Akt signaling pathways, together with RA stimulation, resulted in an extinction of undifferentiated mESCs and a loss of tumorigenicity after grafting into nude mice. This study demonstrates that modulations of Activin A and BMP4 signaling, together with RA stimulation, may be an effective differentiation strategy for eliminating residual tumorigenic ESCs. Moreover, we hypothesized that the ability of pluripotent stem cells to develop teratomas that contain all types of embryonic germ and somatic cell derivatives cells is associated with naїve or germ line states, which have the competency to form germ cells. Consequently, the residual undifferentiated mESCs in teratomas may represent an intermediate cell type similar to naїve pluripotent stem and primordial germ cells, but not to cancerous ECCs, because these cells retained their differentiation potentials to give rise to the derivatives of three germ layers, even after serial transplantations [252]. In contrast, F9 mECCs have significantly restricted differentiation potential and give rise only to the non-tumorigenic cell derivatives after RA stimulation. At the same time, ActivinA could inhibit RA-induced differentiation of multipotent P19 mECCs into neuroectodermal and mesodermal lineages and support the growth of undifferentiated cells [292]. Therefore, the roles of different TGFβ family members in the mechanisms underlying the in vitro and in vivo differentiation of pluripotent stem and teratocarcinoma cells can vary significantly, depending on the configuration of the signal network and the activated transcription factors. However, by modulating the activity and interplay of different signaling pathways, it is possible to regulate the program of differentiation of somatic cells toward the desired directions and to inhibit emerging primordial germ cells, which can initiate the formation of teratomas after transplantation into recipients.

## 6. Conclusions

The TGFβ family members are highly implicated in all the ontogeny steps of pluripotent stem cells, maintaining pluripotent stem cell identity in naїve and primed states, balancing between self-renewal and cell fate signals, initiating differentiation, and specializing lineage derivatives. A significant advance in understanding the TGFβ family signaling-based regulation of these basic cellular processes was achieved using in vitro cell models of pluripotent stem cells, which were obtained from early embryos and by reprogramming somatic cells, as well as ECC lines that were previously isolated from germ cell tumors. Moreover, normal and adapted pluripotent stem and ECC lines are the only well-standardized models for studying the role of TGFβ family signaling in various aspects of cancer stem cell biology: the mechanisms of cancer transformation, cancer evolution and progression, restructuring of signaling pathways and gene regulatory networks, and interactions with various signaling pathways that stabilize normal and abnormal cellular statuses.

Recent studies have improved our understanding of the contribution of the ActivinA/Nodal/Lefty/Smad2/3 and BMP/Smad1/5/8 signaling branches to maintaining pluripotent stem cells in naїve and primed states and to interstate transitions, as well as in switching from self-renewal to lineage commitment. The ActivinA/Nodal/Lefty/Smad2/3 branch was shown to be involved in the regulation of self-renewal and pluripotent stem cell identity through Nanog expression stimulation, whereas the BMP/smad1/5/8 signaling branch demonstrates different contributions to the regulation of proliferation and differentiation of naїve and primed pluripotent stem cells. The final outcome of the activity of each branch is highly dependent on its interaction with the PI3K/Akt, MAPK, and Wnt/Gsk3 signaling pathways and feedback within both branches of the TGFβ family signaling pathway that provide a dynamic equilibrium in the signal network of pluripotent stem cells.

In addition, TGFβ family factors can trigger differentiation in different embryonic and extraembryonic lineages due to the wide repertoire of signaling ligands and their antagonists, dose and time of exposure and interplay with other signaling pathways through common intermediate regulators. High and low level activity of Activin signaling stimulates differentiation of different lineages, into definitive endoderm and mesoderm, respectively [220]. BMP/Smad1/5/8 signaling reduces the expression of pluripotency factors and, in cooperation with Activin/Nodal signaling, triggers endodermal differentiation [170,221], but in the absence of Activin/Nodal and FGF signaling activity, it promotes the differentiation of the trophoblast and extraembryonic endoderm lineages [111,179,218]. Inhibition of both branches of the TGFβ family signaling pathway facilitates the differentiation of neuroectodermal lineages [222]. RSmads-Smad4 complexes, together with different transcriptional partners, activate multiple regulatory sequences of pluripotency and lineage-specific genes and thus provide the possibility of differentiation in various directions, i.e., pluripotency. Crosstalk between the TGFβ family branches and other signaling pathways at each stage of differentiation provides the correct spatiotemporal pattern of cell lineages’ specializations.

Comparative analysis of signaling pathways in ESCs/iPSCs and ECCs revealed different expression patterns of the TGFβ family factors and the activity of the corresponding signaling pathways. The most pronounced difference was found in the activity of the ActivinA/Nodal/Lefty/Smad2/3 branch, whose reduction in ECCs promotes proliferation and inhibits differentiation. Such signaling rearrangements, together with increased activity of the mutant oncogenes Ras and Myc, can lead to the capture of their proliferative status and to complete or partial blockade of differentiation. Multipotent and nullipotent ECCs exist in intermediate states relative to naїve and primed pluripotent states and their differentiation potential is defined by peculiarities in their signal-regulatory networks. Therefore, further studies of TGFβ family signaling pathways and their targets in pluripotent stem cells and ECCs are needed to dissect the functional impairments that lead to the deregulation of proliferation and differentiation in normal and cancerous pluripotent stem cells. Despite significant progress in understanding the biology of pluripotent stem and teratocarcinoma cells, the full picture of the functioning of the signaling pathways and gene regulatory networks in them is still unclear and numerous unresolved issues still warrant further investigation. This knowledge is critical for the development of safe and effective pluripotent stem cell-based technologies for regenerative medicine and new cancer therapies.
